# OMIXCARE: OMICS technologies solved about 33% of the patients with heterogeneous rare neuro-developmental disorders and negative exome sequencing results and identified 13% additional candidate variants

**DOI:** 10.3389/fcell.2022.1021785

**Published:** 2022-10-28

**Authors:** Estelle Colin, Yannis Duffourd, Emilie Tisserant, Raissa Relator, Ange-Line Bruel, Frédéric Tran Mau-Them, Anne-Sophie Denommé-Pichon, Hana Safraou, Julian Delanne, Nolwenn Jean-Marçais, Boris Keren, Bertrand Isidor, Marie Vincent, Cyril Mignot, Delphine Heron, Alexandra Afenjar, Solveig Heide, Anne Faudet, Perrine Charles, Sylvie Odent, Yvan Herenger, Arthur Sorlin, Sébastien Moutton, Jennifer Kerkhof, Haley McConkey, Martin Chevarin, Charlotte Poë, Victor Couturier, Valentin Bourgeois, Patrick Callier, Anne Boland, Robert Olaso, Christophe Philippe, Bekim Sadikovic, Christel Thauvin-Robinet, Laurence Faivre, Jean-François Deleuze, Antonio Vitobello

**Affiliations:** ^1^ Service de Génétique Médicale, CHU d’Angers, Angers, France; ^2^ UFR des Sciences de Santé, GAD “Génétique des Anomalies du Développement”, INSERM-Université de Bourgogne UMR1231, Fédération Hospitalo-Universitaire (FHU)-TRANSLAD, Dijon, France; ^3^ Unité Fonctionnelle Innovation en Diagnostic Génomique des Maladies Rares, Fédération Hospitalo-Universitaire-TRANSLAD, CHU Dijon Bourgogne, Dijon, France; ^4^ Molecular Diagnostics Program and Verspeeten Clinical Genome Centre, London Health Sciences and Saint Joseph’s Healthcare, London, ON, Canada; ^5^ Centre de Génétique et Centre de Référence “Anomalies du Développement et Syndromes Malformatifs”, Hôpital d’Enfants, Centre Hospitalier Universitaire de Dijon, Dijon, France; ^6^ Assistance publique - Hôpitaux de Paris (APHP), Département de Génétique, Groupe Hospitalier Pitié Salpêtrière, Paris, France; ^7^ Service de Génétique Médicale, CHU Nantes, Nantes, France; ^8^ Sorbonne Université/INSERM U1127/CNRS UMR 7225/Institut du Cerveau, Paris, France; ^9^ Service de Neurologie, Hôpital la Pitié Salpêtrière, Sorbonne Université, Paris, France; ^10^ Département de Génétique, Assistance publique - Hôpitaux de Paris Sorbonne Université, Hôpital Pitié-Salpêtrière et Trousseau, Paris, France; ^11^ Assistance publique - Hôpitaux de Paris, Département de Génétique, Sorbonne Université, GRC No. 19, ConCer-LD, Centre de Référence Déficiences Intellectuelles de Causes Rares, Hôpital Armand Trousseau, Paris, France; ^12^ Service de Génétique Clinique, European Reference Network (ERN) ITHACA, CHU Rennes, Rennes, France; ^13^ IGDR (Institut de Génétique et Développement de Rennes)—UMR 6290, ERL U1305, CNRS, INSERM, Univ Rennes, Rennes, France; ^14^ Service de Génétique Médicale, CHU de Tours, Tours, France; ^15^ Commissariat à l'énergie atomique et aux énergies alternatives (CEA), Centre National de Recherche en Génomique Humaine (CNRGH), Université Paris-Saclay, Evry, France; ^16^ LabEx GENMED (Medical Genomics) Paris France; ^17^ Department of Pathology and Laboratory Medicine, Western University, London, ON, Canada; ^18^ Centre de Référence Maladies Rares “Déficiences Intellectuelles de Causes Rares”, Centre de Génétique, Fédération Hospitalo-Universitaire-TRANSLAD, CHU Dijon Bourgogne, Dijon, France

**Keywords:** undiagnosed neurodevelopmental diseases, genome sequencing, transcriptome sequencing, DNA methylation analysis, translational research

## Abstract

**Purpose:** Patients with rare or ultra-rare genetic diseases, which affect 350 million people worldwide, may experience a diagnostic odyssey. High-throughput sequencing leads to an etiological diagnosis in up to 50% of individuals with heterogeneous neurodevelopmental or malformation disorders. There is a growing interest in additional omics technologies in translational research settings to examine the remaining unsolved cases.

**Methods:** We gathered 30 individuals with malformation syndromes and/or severe neurodevelopmental disorders with negative trio exome sequencing and array comparative genomic hybridization results through a multicenter project. We applied short-read genome sequencing, total RNA sequencing, and DNA methylation analysis, in that order, as complementary translational research tools for a molecular diagnosis.

**Results:** The cohort was mainly composed of pediatric individuals with a median age of 13.7 years (4 years and 6 months to 35 years and 1 month). Genome sequencing alone identified at least one variant with a high level of evidence of pathogenicity in 8/30 individuals (26.7%) and at least a candidate disease-causing variant in 7/30 other individuals (23.3%). RNA-seq data in 23 individuals allowed two additional individuals (8.7%) to be diagnosed, confirming the implication of two pathogenic variants (8.7%), and excluding one candidate variant (4.3%). Finally, DNA methylation analysis confirmed one diagnosis identified by genome sequencing (Kabuki syndrome) and identified an episignature compatible with a BAFopathy in a patient with a clinical diagnosis of Coffin-Siris with negative genome and RNA-seq results in blood.

**Conclusion:** Overall, our integrated genome, transcriptome, and DNA methylation analysis solved 10/30 (33.3%) cases and identified a strong candidate gene in 4/30 (13.3%) of the patients with rare neurodevelopmental disorders and negative exome sequencing results.

## 1 Introduction

Rare and ultra-rare genetic diseases, defined as having an average global prevalence of 1 in 2,500 and 1 in 50,000, respectively, collectively affect about 350 million of the general population (Ferreira, 2019). Affected individuals and their families experience a diagnostic odyssey lasting on average 5 years (“Global Commission | Ending the Diagnostic Odyssey for Children with a Rare Disease” n.d.). However, early molecular diagnosis is fundamental for a better understanding of the disease, informed care in general medicine, and genetic counseling. Over the past decade, high-throughput sequencing, and in particular whole exome sequencing (ES), which enriches coding regions, representing ∼1.5% of the human genome, has rapidly become the first-line genomics assay in clinical settings. Its diagnostic yield ranges from 30% to 50% in patients presenting with heterogeneous rare syndromic genetic disorders with suspected Mendelian inheritance (McInerney-Leo et al., 2013; Veeramah et al., 2013; Clark et al., 2018). However, molecular diagnosis remains elusive in 50%–75% due to 1) the challenge of interpreting the data, 2) technological limitations [i.e., mosaic variants, repeat expansions, or structural variants (SVs) not correctly detected through ES], 3) non-coding regulatory variants affecting promoters, enhancers, deep intronic regions, or distant-acting regulatory sequences located in intergenic regions, and 4) complex inheritance (Frésard and Montgomery, 2018; Boycott et al., 2019; Hartley et al., 2020).

There is growing interest in whole genome sequencing (GS) coupled with total RNA sequencing (RNA-seq) in translational research settings. Indeed, GS explores variants in the coding and non-coding regions with fewer technological limitations although the challenge of interpreting the variants remains. GS analysis detects more than three million single nucleotide variants (SNV) and more than 1,500 SVs per individual on average. Of these three million SNVs, 30,000 are rare, and some are expected to have a significant impact on gene expression or alternative splicing. RNA-seq is able to measure variations in RNA abundance, allele-specific expression, and aberrant splicing, which assists with interpretation of variants. Thus, some recent studies reported an increased diagnostic yield of 7.5%–35% using RNA-Seq as a complementary approach to ES or GS in well-defined diseases, with homogeneous cohorts of patients and appropriate sample tissues (Cummings et al., 2017; Kremer et al., 2018; Frésard et al., 2019; Gonorazky et al., 2019; Hamanaka et al., 2019; Lee et al., 2020; Murdock et al., 2020; Stenton and Prokisch, 2020; Yépez et al., 2022). Furthermore, the study of genome-wide DNA methylation profiles in peripheral blood as biomarkers associated with rare developmental disorders has been demonstrating its utility for the assessment and the reclassification of variants of unknown significance in diagnostic settings (Aref-Eshghi et al., 2019; Aref-Eshghi et al., 2020; Sadikovic et al., 2021; Levy et al., 2022).

In this context, our project aimed to integrate short-read genome sequencing, messenger RNA-seq analysis, and methylation studies as complementary translational research tools to examine several individual-derived samples and look for rare diseases associated with neuro-developmental disorders, when the first line and high-quality trio ES had produced negative results.

## 2 Materials and methods

### 2.1 Recruitment of individuals and data sharing

Thirty individuals were recruited from four genetics centers belonging to the French network for rare diseases (CHU Dijon, CHU Nantes, CHU Rennes, APHP Paris) and carefully evaluated by our interdisciplinary clinical-biological team. Affected individuals with malformation syndromes and/or severe neurodevelopmental disorders, with negative trio exome sequencing and array comparative genomic hybridization results were enrolled. Informed consent was obtained from all subjects participating in the study.

### 2.2 DNA extraction—quantity and quality controls

DNA was extracted from blood collected in EDTA tubes. 3–5 ml of whole blood was incubated for 10 min in RBC lysis buffer (Qiagen GmbH, Hilden, Germany) and then centrifuged for 2 min at 2000 rpm to obtain white blood cell pellet, which was resuspended in 180 µl of residual supernatant and 20 µl of RNAse A (Qiagen GmbH, Hilden, Germany). Purification was then performed using the QiAamp DNA Blood mini kit on a QiaCube extraction device following the standard protocol.

Quantification was obtained using the Qubit dsDNA HS Assay (Life Technologies, CA, United States) and gel electrophoresis. The purity of DNA was verified through an evaluation of the 260/280 and 260/230 absorbance ratios on a Multiskan Go device (Thermo Scientific, Waltham, MA, United States).

At least 4 µg of DNA was needed per sample to use for quality control before sequencing at the CNRGH platform and to potentially prepare a second library in the event of technical problems. If the quantity or quality of DNA from a sample was insufficient, a new sample was requested from the center.

### 2.3 RNA extraction—quantity and quality control

Total RNA was extracted from whole blood collected in PAXgene tubes (Preanalytics GmbH, Hombrechtikon, Switzerland) using the PAXgene Blood RNA kit (Preanalytics GmbH, Hombrechtikon, Switzerland) automated on a QiaCube extraction device (Qiagen GmbH, Hilden, Germany) following the standard protocol. Alternatively, RNA was extracted from fibroblast cell cultures using TRIzol^®^ RNA isolation reagent (ThermoFisher).

RNA was then quantified by measuring absorbance using a NanoDrop device. The quality was assessed by determining the RNA Integrity Number (RIN) on the bioanalyzer device (Agilent Technologies, Santa Clara, CA, United States). RNA was suitable for RNA-Seq if the RIN was at least 7.

### 2.4 Short-read genome sequencing

The genomic DNA libraries were prepared following the TruSeq DNA PCR-free protocol (Illumina, CA, United States). A minimum of 1 µg of genomic DNA was sheared by sonication and then purified. Oligonucleotide adaptors to sequence both ends were ligated on end-repaired fragments and then purified. DNA libraries were barcoded (indexed) and then multiplexed. GS was performed at the *Centre National de Recherche en Génomique Humaine* (CNRGH, CEA) using the Illumina NovaSeq6000 platform (Illumina, CA, United States), generating 150 base pairs paired-end reads. Data sequencing was required to meet minimum quality standards, with an average of over ×35 depth of coverage and more than 97% of the genome covered by at least 10 reads.

### 2.5 RNA sequencing

RNA-seq sequencing was performed by the CNRGH (CEA). After complete RNA quality control (quantified in duplicate on a NanoDrop™ 8,000 spectrophotometer and RNA 6000 Nano LabChip analysis on a Bioanalyzer from Agilent), libraries were prepared using the TruSeq Stranded mRNA Library Prep Kit (Illumina). All libraries were prepared on an automated platform using an input of 1 µg of total RNA, in line with the manufacturer’s instructions. Library quality was checked on a LabChip GX (Perkin Elmer) for profile analysis and quantification, and sample libraries were pooled before sequencing, to reach the expected sequencing depth. Sequencing was performed on an Illumina HiSeq 4,000 as paired-end 100 bp reads, using dedicated Illumina sequencing reagents. Libraries were generally pooled using four samples per lane. FASTQ files produced after RNA-seq sequencing were then processed by in-house CNRGH tools to assess the quality of raw and aligned nucleotides.

### 2.6 DNA methylation data analysis

Methylation analysis was performed with version 3 of the clinically validated EpiSign™ assay as previously described (Aref-Eshghi et al., 2020, 2019; Sadikovic et al., 2021; Levy et al., 2022).

### 2.7 Bioinformatics analysis

#### 2.7.1 Short-read genome sequencing

Variants were identified using the FHU Translad computational platform, hosted by the University of Burgundy Computing Cluster (CCuB). Raw data quality was evaluated by FastQC software (v0.11.4). Reads were aligned to the GRCh37/hg19 human genome reference sequence using the Burrows-Wheeler Aligner (v0.7.15) and subsequently to GRCh38 for reanalysis. Aligned read data underwent the following steps: 1) duplicate paired-end reads were removed by Picard software (v2.4.1), and 2) base quality score recalibration was done by the Genome Analysis Toolkit (GATK v3.8) Base recalibrator. Using GATK Haplotype Caller, Single Nucleotide Variants with a quality score >30 and an alignment quality score >20 were annotated with SNPEff (v4.3). Rare variants were identified by focusing on nonsynonymous changes at a frequency of less than 1% in the gnomAD database.

Copy Number Variants were detected using two approaches: the first based on read depth analysis using Control-FREEC (v11.4) and the second on anomalous read pairs combined with split-read detection using Lumpy (v0.2.12). The resulting CNVs and SVs were annotated using in-house python scripts and were filtered in terms of their frequency in public databases (DGV, ISCA, DDD).

#### 2.7.2 RNA-sequencing

Aberrant splice events and expression outliers were identified using the FHU Translad computational platform, hosted by the University of Burgundy Computing Cluster (CCuB). Raw data quality was evaluated by FastQC software (v0.11.4). Reads were aligned to the GRCh37/hg19 human genome reference sequence using the STAR2 Aligner (v2.5.2b) with the 2-pass mapping method using the human RefSeq genome annotation (Build GCF_000001405.25). Read counts were also collected using STAR2. Uniquely mapped reads are counted when overlapping only one gene.

Outlier expressed genes were detected using two parallel methods: DESeq2 (v1.26.0) and Outrider (v1.4.2). After a normalization step, the expression analysis was performed using the following analysis design: one versus the whole analysis batch, allowing computation of the expression variance for the whole cohort. A Z-score was computed, and filters were applied to only keep genes with a z-score superior to 3 or inferior to −3.

Aberrant splice events were detected using three parallel methods: rMATS (v4.0.2), LeafCutter (v0.2.9), and a custom method derived from Cummings et al. (2017)

rMATS allowed us to compute a Percent Spliced In (PSI) value, indicating the proportion of the junction involved in a splice event. LeafCutter performs an intron analysis using a clustering method. For both methods, a Z-score was computed and the same filters were applied as for expression. The custom method considered each splice junction as a rare variant and applied a filter based on frequency in the cohort to select only rare events.

#### 2.7.3 DNA methylation data analysis

Briefly, methylated and unmethylated signal intensity generated from the EPIC array was imported into R 3.5.1 for normalization, background correction, and filtering. Beta values ranging from 0 (no methylation) to 1 (complete methylation) were calculated as a measure of methylation level and processed through the established support vector machine (SVM) classification algorithm for EpiSign disorders. The EpiSign Knowledge Database, composed of over 10,000 methylation profiles from reference disorder-specific and unaffected control cohorts, was used by the classifier to generate disorder-specific methylation variant pathogenicity (MVP) scores. MVP scores represent confidence of prediction for each disorder, ranging from 0 (discordant) to 1 (highly concordant). A positive classification typically generates MVP scores greater than 0.5. These scores, in combination with the assessment of hierarchical clustering and multidimensional scaling, are used in generating the final matched EpiSign result.

## 3 Results

### 3.1 Characteristics of the cohort

**FIGURE 1:**
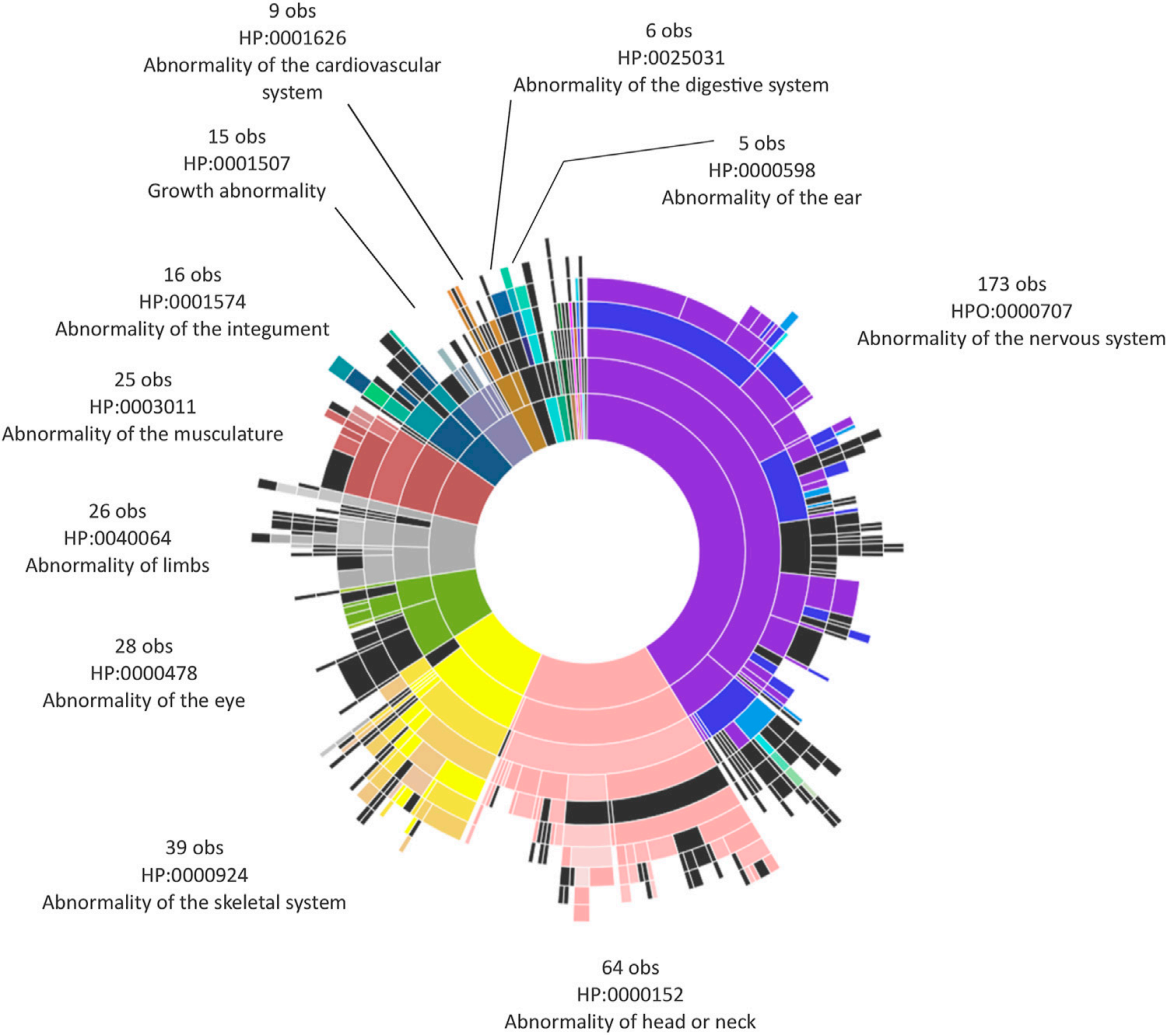
Human Phenotype Ontology (HPO) terms observed in the cohort. Sunburst plot depicting the hierarchical organization of ontologies described in our cohort, based on the Human Phenotype Ontology (http://purl.obolibrary.org/obo/hp.obo; format-version: 1.2; data-version: hp/releases/2019-02-12). The phenotypic abnormalities, representing the roots or the topmost terms in the hierarchy, are depicted as semi-circular sections at the center of the sunburst. For each phenotypic abnormality and its corresponding HP code, the number of observations stemming from each root is reported. The Sunburst plot was obtained using the JavaSript library D3.js—https://d3js.org

The cohort was mainly composed of pediatric individuals (22/30; 73%), and the sex distribution was mostly female (19/30; 63%). Only two individuals (6%) came from consanguineous unions. The median age of our cohort was 13.7 years (4 years and 6 months to 35 years and 1 month), including eight adult patients aged 18–35 years and 1 month. Phenotypic data were collected as Human Phenotype Ontology (HPO) terms. For each individual, at least two HPO terms and at most 11 HPO terms were collected, giving rise to a global dataset of 417 observations (Figure 1). The most represented terms, accounting for 66% of the available HPO terms, included abnormalities of the nervous system (41.3%), head and neck (15.3%), and skeletal system (9.3%). Clinical data of the individuals are available in Supplementary Table S2 and Supplementary Data.

### 3.2 Diagnostic rate of genome sequencing

**FIGURE 2:**
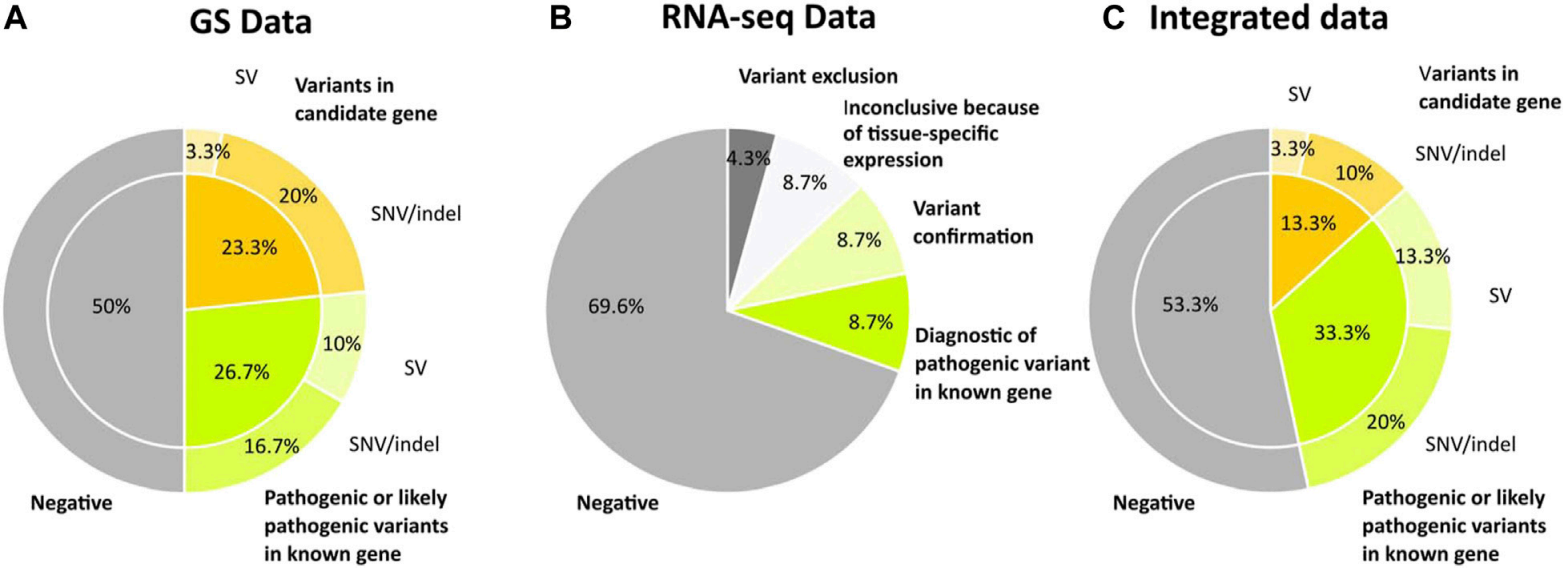
Diagnostic yield obtained with the different approaches deployed. Schematic representation of the evolution of the diagnostic yield in our cohort of 30 individuals with heterogeneous rare neurodevelopmental disorders. **(A)** The initial diagnostic yield with the genome sequencing (GS) data alone. **(B)** Contribution of RNA sequencing (RNA-seq) to the diagnostic rate in 23/30 patients. **(C)** Diagnostic yield obtained by integrating GS, RNA-seq and DNA methylation results. GS, genome sequencing; RNA seq, RNA sequencing; SV, structural variant; SNP, single nucleotide variants; indel, insertion deletion.

In eight out of 30 individuals (26.7%), we identified at least one causative variant [class 4 or 5 of ACMG Guidelines (Richards et al., 2015)]. These included three single nucleotide variants (SNP) and three indels: a missense variant in *CYFIP2* in individual 9, a nonsense variant in *KMT2D* for individual 6 and in *TMEM147* for individual 12, and frameshift variants in *FOXG1*, *PURA* and *TMEM147* in individuals 7, 8, and 12, respectively. Three SVs were identified: one intragenic heterozygous deletion-inversion of 9.4 kb in *CASK* in individual 1, one partial intragenic heterozygous deletion of 37 kb in *GATAB2D* in individual 2, and one heterozygous balanced inversion of about 2.2 Mb of a regulatory region of *MEF2C* in individual 3 (Table 1; Figure 2). The variants in *PURA*, *KMT2D*, and *FOXG1* had not been identified by ES because the capture kits utilized did not cover these regions. All these variants occurred *de novo* but the *TMEM147* variants followed a recessive mode of inheritance. Furthermore, *TMEM147* was initially identified as a new candidate gene, and data sharing and functional studies allowed us to confirm its causal role (Thomas et al., 2022).

**TABLE 1:** Causative, candidate and excluded candidate genes of the cohort SNV, single nucleotide variant; indel, insertion-deletion; SV, structural variant. GRCh37-hg19 Genome Reference Consortium Human Build 37,NM_ c./r. Human Genome Variation Society nomenclature at the transcript or the RNA level p. nomenclature at the transcrip level ACMG American College of Medical Genetics and Genomics OMIM Online Mendelian Inheritance in Man.

	Gene Individual	(GRCh37—hg19) g.	(NM_) c./r.	p.	ACMG class	Inheritance	OMIM
Positive results							
SNV/indel	*KMT2D Individual 6*	NC_000012.11:g.49426598G>A	NM_003482.3: c.11890C>T	p.(Gln3964*)	5	*De novo*	Kabuki syndrome **#** 147920
	*FOXG1 Individual 7*	NC_000014.8:g.29236741dup	NM_005249.4: c.256dup	p.(Gln86Profs*35)	5	*De novo*	Rett syndrome **#** 613454
	*PURA Individual 8*	NC_000005.9:g.139493864dup	NM_005859.4: c.98dup	p.(Gly34Argfs*167)	5	*De novo*	Mental retardation, autosomal dominant 31 # 616158
	*CYFIP2 Individual 9*	NC_000005.9:g.156754997A>G	NM_014376.2: c.2096A>G	p.(Asp699Gly)	5	*De novo*	Developmental and epileptic encephalopathy 65 # 618008
	*TCF4 Individual 30*	NC_000018.9:g.52926128C>T	NM_001083962.2:c.1069+1052G>A NM_001083962.2:r.1069_1070ins[1069+833_1069+1,049]	p.(Ala357Glyfs*7)	5	*De novo*	Pitt-Hopkins syndrome # 610954
	*TMEM147 Individual 12*	NC_000019.9:g.36036812_36036830del NC_000019.9:g.36038077C>G	NM_032635.3: c.100_118delc.486C>G	p.(Lys34Serfs*33) p.(Tyr162*)	5	Recessive mode of inheritance	*613585
SV	*CASK Individual 1*	NC_000023.10: g.41387135_41396533delins41391688_41391989inv	NM_003688.3:r.2156_2505del	p.(Asp719Glyfs*28)	5	*De novo*	Mental retardation, with or without nystagmus **#** 300422
	*GATAD2B Individual 2*	NC_000001.10: g.[?_ 153753742)_( 153791156 _?]del			5	*De novo*	GAND syndrome **#** 615074
	*SPTAN1 Individual 5*	NC_000009.11: g.[?_ 131382516)_( 131393966 _?]del	NM_001130438.2:r.5734_6762del	p.(Gly1912_Lys2254del)	5	*De novo*	Developmental and Epileptic Encephalopathy 5 **#** 613477
	*MEF2C Individual 3*	NC_000005.9:g.[88625547_88635553delinsTA; 88635554_90795688inv; 90795689_90795690del]			3	*De novo*	Mental retardation, stereotypic movements, epilepsy, and/or cerebral malformations **#** 613443
Candidate							
SNV/indel	*POLA1 Individual 10*	NC_000023.10:g.25013973A>T	NM_016937.3 c.4295A>T	p.(Lys1432Ile)	3	Maternal	Van Esch-O’Driscoll **#** 301030
	*ARI5B Individual 13*	NC_000010.10:g.63845563del	NM_032199.2 c.1302del	p.(Asn434fs)	3	*De novo*	* 608538
	*GRIN2B Individual 14*	NC_000012.11:g.13893083A>G	NM_000834.3 c.1010+13168T>C	p.?	3	*De novo*	Intellectual developmental disorder, autosomal dominant 6, with or without seizures **#** 613970
SV	Chromoanagenesis *Individual 4*	Complex rearrangement involving chromosomes 6 and 11			3	*De novo*	
Excluded candidate							
SNV/indel	*SENP6 Individual 15*	NC_000006.11:g.76350410C>T	NM_015571.2 c.469C>T	p.(Arg157*) p.(Arg157*)	2	Recessive mode of inheritance	
	*FGD1 Individual 11*	NC_000023.10:g.54492281G>A	NM_004463.3 c.1345C>T	p.(Arg449Cys)	2	Maternal	Intellectual developmental disorder, X-linked, syndromeic 16 **#** 305400

We also identified at least a candidate disease-causing variant in seven additional individuals (23.3%). Six SNVs, including a hemizygous missense variant in *POLA1* in individual 10 and in *FGD1* in individual 11, both inherited from healthy mothers, a homozygous nonsense variant in *SENP6* in individual 15, two *de novo* deep intronic non-coding variants in *GRIN2B* and *TCF4* in individuals 14 and 30 respectively, and one *de novo* indel in *ARI5B* in individual 13 were identified. Furthermore, *de novo* complex structural variants involving two chromosomes (i.e., chromoanagenesis) were identified in individual 4 (Table 1; Figure 2). Data sharing allowed us to corroborate the suspected involvement of the *de novo ARI5B* variant in individual 13. Clinical and molecular data of the individuals are available in the Supplementary Data.

### 3.3 Diagnostic rate from RNA sequencing data

**FIGURE 3:**
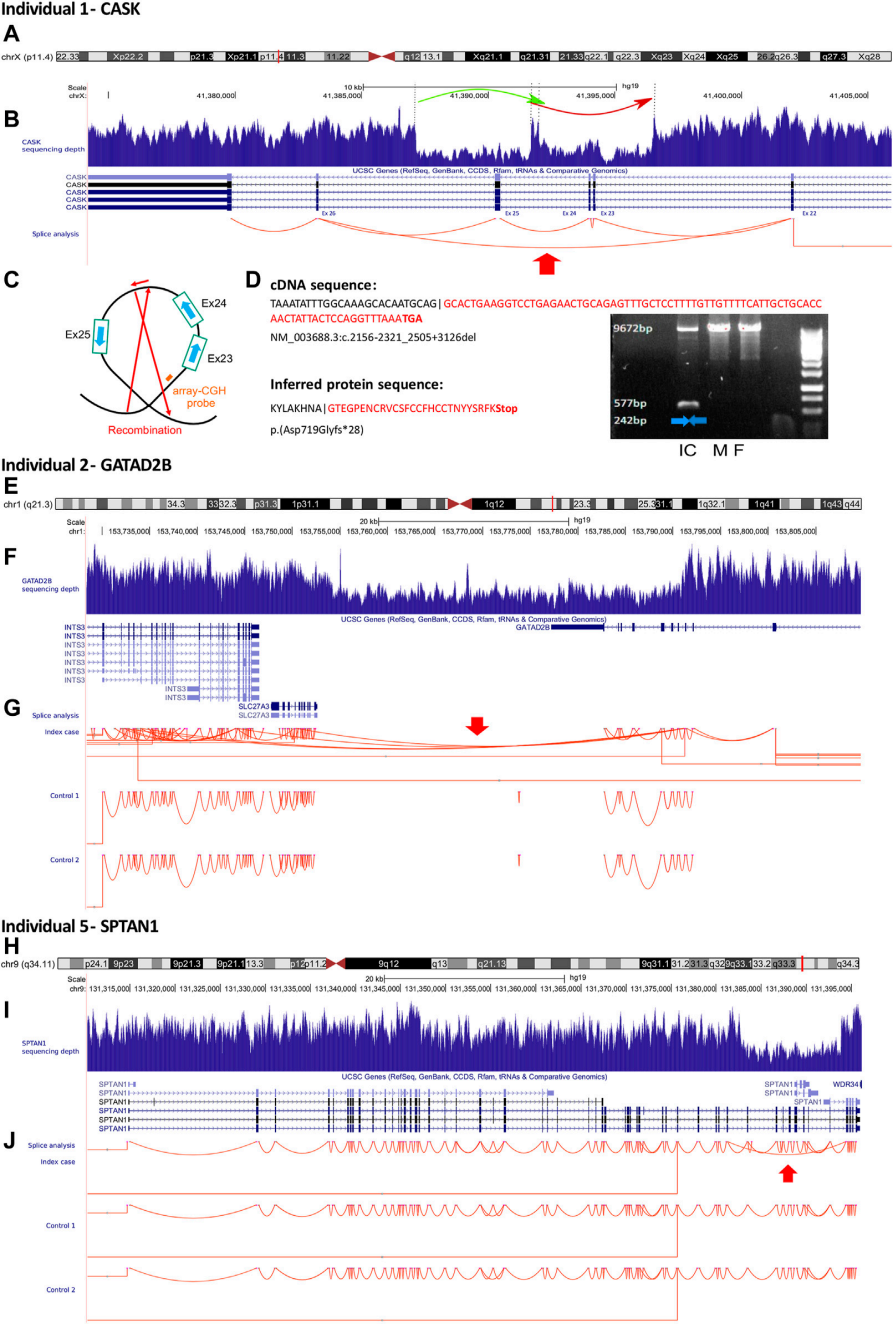
Illustration of individuals 1—CASK, 2—GATAD2B, and 5—SPTAN1. **(A)** Ideogram showing chromosome X and *CASK* localization. Under the ideogram, the green and red arrows represent the deletion-inversion of 9.4 kb in Xp11.4. **(B)** UCSC genome browser snapshot with visualization of *CASK* sequencing depth and *CASK* splice analysis. *CASK* sequencing depth demonstrates an intragenic deletion encompassing exons 23 through 25. The red arrow in the splicing analysis demonstrates the presence of a heterozygous transcript lacking exons 23 through 25. **(C)** Graphic illustrating the mechanism of the loss of three exons from the *CASK* gene. The deletion was not detected by array CGH as the deleted region contained only one probe. **(D)** PCR confirmed deletion in proband but this was absent in two controls. Splice reads defined the readout of the event, identifying a frameshift variant. **(E)** Ideogram showing chromosome 1 and *GATAD2B* localization **(F)** UCSC genome browser snapshot showing sequencing depth, and **(G)** splice analysis at the *GATAD2B* locus. The sequencing depth shows a partial deletion of ∼37 Kb of *GATAD2B* encompassing exons 5 through 11. The red arrow indicates the presence of a heterozygous transcript showing a fusion transcript between *GATAD2B* and *SLC27A3*. **(H)** Ideogram showing chromosome 9 and *SPTAN1* localization. **(I)** UCSC genome browser snapshot with visualization of sequencing depth and **(J)** splicing analysis at the *SPTAN1* locus. The sequencing depth shows the partial deletion of ∼11 kb of *SPTAN1* including exons 44 through 51. The red arrow indicates the presence of a heterozygous transcript showing exon skipping in *SPTAN1*.

RNA-seq from whole blood was performed in 23 individuals (76.3% of the cohort): 11 undiagnosed individuals, eight with candidate genes, and five with positive GS. For the remaining seven individuals (23.3%), RNA-seq was not performed either because GS alone had already identified the causative variant (*KMT2D*, *PURA*, *CYFIP2*) or because the RNA was not available or did not pass the quality control standards (RIN ≥ 7). RNA-seq analysis confirmed the causal role of two variants in *CASK* and *GATAD2B* (2/23; 8.7%). In particular, an aberrant splicing event was found in individual 1, who harbored a *de novo* deletion-inversion of 9.4 kb in Xp11.4 involving *CASK*, while the partial deletion of *GATAD2B* was identified in RNA-seq data as an expression outlier due to nonsense-mediated mRNA decay, accompanied by gene expression down-regulation. RNA-seq also led to the identification of one additional diagnosis consisting of a *de novo* deletion in *SPTAN1* of about 11 kb, not detected by our CNV pipeline, associated with a splicing anomaly (Figure 3). The blood RNA-seq data from individual 30 did not allow us to confirm the pathogenic effect of the *de novo* intronic variant in *TCF4*, which was predicted to create a donor splice site. Indeed, *TCF4* expression was barely detectable in blood-derived RNA-seq data. However, we also obtained a fibroblast cell culture from the same patient, and the RNA-seq data from this sample revealed the retention of a cryptic exon of 218 nt, causing a frameshift variant. The nonsense-mediated decay of the transcript carrying the cryptic exon was supported by the observation of a skewed allelic expression of an informative polymorphism in the 3′ end of the transcript (Supplementary Figure S1). The *TCF4* gene is responsible for Pitt-Hopkins syndrome, which is characterized by intellectual disability, wide mouth and distinctive facial features, and intermittent hyperventilation followed by apnea (MIM 602272) (Amiel et al., 2007). Reverse phenotyping was consistent with this syndrome.

Overall, RNA-seq identified two additional diagnoses (2/23; 8.7%) and independently confirmed two pathogenic variants already identified by GS (i.e., *CASK* and *GATAD2B*) (2/23; 8.7%). RNA-seq also allowed us to exclude the candidate variant in *SENP6* (1/23; 4.3%). Indeed, *SENP6* was not identified as a transcriptome outlier as the RNA-seq did not show any significant down-regulation of this gene, indicating that the nonsense variant p.(Arg157*) affected a minor isoform. These results were corroborated by a more accurate analysis of GTEx data, revealing that the RefSeq transcript NM_015571.4, corresponding to the MANE select transcript ENST00000447266.7 is ranked third in terms of abundance in all tissues and in particular in the central nervous system. Furthermore, this exon was also alternatively spliced in the computed GTEx gene model. Finally, RNA-seq did not show any monoallelic expression secondary to the *MEF2C* regulatory inversion or an aberrant splice event in *GRIN2B* in blood and fibroblast cell lines because the respective genes showed a neural-specific expression (2/23; 8.7%) (Figure 2), hence the analysis remained inconclusive for these variants. Splicing and expression abnormalities were validated by visual inspection of the RNA-seq alignment in the Integrative Genomics Viewer (IGV) (Robinson et al., 2017).

### 3.4 Analysis of DNA methylation profiles

DNA methylation profiles from whole blood were performed for all individuals from the same sample as was used for GS. EpiSign™ analysis revealed a genome-wide DNA methylation profile consistent with one of the 59 established episignatures in 10% (3/30) of cases assessed. All positive cases obtained a high confidence methylation variant pathogenicity (MVP) score of 1.0 (Supplementary Figure S2) with supportive multidimensional scaling (MDS) and hierarchical clustering. The patients positive for Episign episignatures were: individual 6 with a molecular diagnosis of Kabuki syndrome made by GS analysis (Kabuki syndrome due to variants in *KMT2D* or *KDM6A*), individual 11 with a clinical diagnosis of Coffin-Siris and negative GS and blood RNA-seq results (BAFopathy due to variants in *ARID1A*, *ARID1B*, *SMARCB1*, *SMARCA2* or *SMARCA4*), and individual 13 with a *de novo* variant in *ARID5B* (Wolf-Hirschhorn syndrome caused by deletions at 4p16.3). Interestingly, for individual 11, DNA methylation analysis also allowed us to exclude the implication of the variant of unknown significance (VUS) in *FGD1* identified by GS analysis. Furthermore, the analysis of genes involved in BAFopathies did not reveal any aberrant hypermethylation at promoters or gene body regions (Supplementary Figure S3). The visual inspection of genes involved in BAFopathies did not reveal any obvious structural variants. In addition, in individual 13, whose *ARID5B* candidate variant was found by GS, reverse phenotyping was not consistent with Wolf-Hirschhorn syndrome, and nor was a deletion in 4p16.3 found by array CGH and GS, suggesting that *ARID5B* mutation may share some molecular biomarkers in common with Wolf-Hirschhorn syndrome.

Finally, two cases were inconclusive for the episignatures for Velocardiofacial syndrome (individual 23) and Rubinstein-Taybi syndrome (individual 25), as MVP elevation (<0.5) was insufficient and MDS and hierarchical clustering were inconsistent. Reverse phenotyping in individual 23 was not consistent with Velocardiofacial syndrome. Indeed, this individual showed a severe intellectual disability with microcephaly, myoclonic absence seizure, and hypoplasia of the corpus callosum. However, the clinical diagnostic hypothesis for individual 25 was Coffin-Siris syndrome.

All in all, we were able to diagnose ten out of 30 individuals (33.3%) and to have a candidate gene in four out of 30 individuals (13.3%) (Figure 2; Table 1; Supplementary Table S1).

### 3.5 Illustrative cases

#### 3.5.1 Individual 1—*CASK*

Individual 1 was a 7-year-old girl, the only child of unaffected, non-consanguineous French parents. The pregnancy had been uncomplicated. She was born at 39 WG with normal birth length (48.5 cm, p37), weight (3290 g, p58), and OFC (32.5 cm, p11). The neonatal period was marked by poor feeding. All motor development milestones were delayed: she was able to sit independently at 9.5 months and walk at 2 years of age. She presented with delayed speech and language development. A brain MRI was performed and it was normal. Physical examination revealed no obvious dysmorphic features or microcephaly (−3.5 SD). She presented with bruxism. Previous genetic investigations, consisting of array CGH and trio ES, had been normal. GS identified a rearrangement of the *CASK* gene. This was a *de novo* deletion-inversion of 9.4 kb in Xp11.4 (Figure 3). RNA-seq identified an aberrant splicing event involving exons 23 through 25 skipping. This deletion was verified in qPCR. The *CASK* gene is involved in X-linked dominant intellectual disability with or without nystagmus (MIM 300422).

#### 3.5.2 Individual 2—*GATAD2B*

Individual 2 was a 24-year-old male, the child of unaffected, non-consanguineous French parents. The pregnancy had been marked by ventriculomegaly at 22 WG. He was born at 41 WG with normal birth length (50.5 cm, p37) and weight (3040 g, p8), and macrocephaly with an OFC of 37.2 cm, (p92). During the neonatal period, he presented with hypotonia and poor feeding, followed by global developmental delay with language impairment and severe intellectual disability. Brain MRI was normal. His facial dysmorphisms included macrocephaly, prominent forehead, hypertelorism, and small, low-set ears. Physical examination revealed long toes, finger swelling and excessive wrinkling of palmar skin. He experienced hyperactivity in infancy, and subsequently short attention span, restricted behaviors, and sleep disturbance. Previous genetic investigations, including array CGH, screening for Sotos syndrome (*NSD1*) and Cowden syndrome (*PTEN*), intellectual disability panel, and trio ES, had returned normal results. GS identified a *de novo* partial deletion of ∼37 kb of the *GATAD2B* gene with breakpoints within two AluY elements flanking the deleted region (Figure 3). This deletion was confirmed by a high-resolution array CGH but had not been identified by the first array CGH because of the lack of probes in this region. Transcriptome outlier detection confirmed the partial deletion of *GATAD2B*. The *GATAD2B* gene is responsible for the neurodevelopmental syndrome GAND, which combines hypotonia, psychomotor retardation, language disorders, intellectual disability, macrocephaly, and shared facial features (MIM. 615074). Reverse phenotyping was consistent with GAND syndrome.

#### 3.5.3 Individual 3—*MEF2C*

**FIGURE 4:**
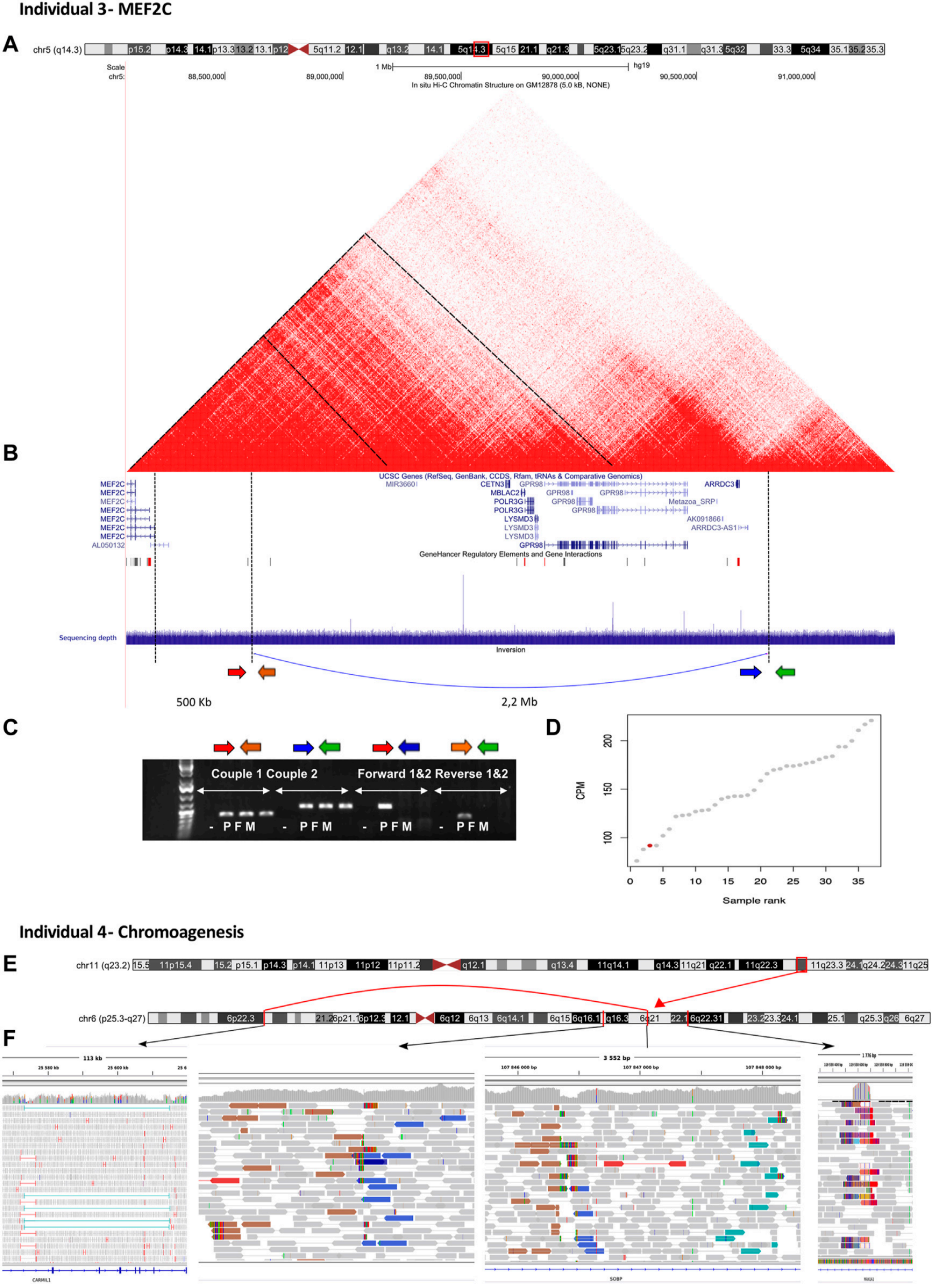
Illustration of individual 3—MEF2C and individual 4—Chromoanagenesis. **(A)** Ideogram showing chromosome 5 and *MEF2C* localization. **(B)** UCSC genome browser snapshot with visualization of three-dimensional (3D)-genome map at the 5q14.3 locus derived from Hi-C data of gM1287825 (10 kb resolution) and sequencing depth of this region. The structural variant characterized by a *de novo* inversion of 2.2 Mb in 5q14.3 is shown in blue under the sequencing depth track. It encompasses part of the regulatory region responsible for the neuronal expression of the *MEF2C* gene. The inversion did not include the gene body as it was located 500 Kb away from its proximal promoter region. This inversion is expected to deregulate *MEF2C via* its topologically associating domain dysfunction. **(C)** PCR analysis confirming the inversion. **(D)** *MEF2C* expression with RNA sequencing data. There was low expression of the MEF2C transcript in the individual; however, its biallelic expression in blood suggested that the regulatory regions affected by this inversion were specific to the neuronal lineage. **(E)** Ideogram showing chromosomes 11 and 6 affected by the complex rearrangement. **(F)** IGV visualization of the breakpoint located in chromosome 6.

Individual 3 was an 11-year-old girl, the second child of unaffected, non-consanguineous French parents. The pregnancy was uncomplicated. She was born at 38 WG with intrauterine growth retardation, birth length 45.5 cm (p7), birth weight 2630 g (p16), and OFC of 34.5 cm (p71). All motor development milestones were delayed: she was able to sit independently at 19 months, and was still unable to walk at 11 years of age. She presented with language impairment and behavioral problems such as abnormally aggressive, impulsive or violent behavior. A first EEG at 11 months of age showed some spike-wave discharges. At 8 years of age, EEG showed typical absence seizures. A brain MRI showed enlargement of the pericerebral spaces and slight hyperintensity of posterior cerebral white matter. She had facial dysmorphisms, including a prominent forehead, deep philtrum, and wide mouth with full lips. Previous genetic investigations, consisting of array CGH, screening for Angelman syndrome (methylation and sequencing of *UBE3A*) and Fragile X Syndrome (*FMR1*), sequencing of *FOXG1*, *CDKL5*, *STK9*, *RAI1*, *MECP2*, *MEF2C*, and trio ES, had returned normal results. GS identified a pathogenic structural variant characterized by a *de novo* inversion of 2.2 Mb in 5q14.3 encompassing part of the regulatory region responsible for the neuronal expression of the *MEF2C* gene. This rearrangement was confirmed on qPCR. MEF2C expression with RNA sequencing data showed low expression of the MEF2C transcript in this individual, although there was biallelic expression in blood, confirming that the regulatory regions affected by the inversion were specific for the neuronal lineage (Figure 4). The *MEF2C* gene is responsible for neurodevelopmental disorders with hypotonia, stereotypic hand movements, and impaired language (MIM 613443). Reverse phenotyping was consistent with this diagnosis.

#### 3.5.4 Individual 4—chromoanagenesis

Individual 4 was a 15-year-old boy, the first child of unaffected, non-consanguineous French parents. The pregnancy had been uncomplicated. He was born at 41 WG with normal birth length (52 cm, p70) and weight (3700 g, p60), and macrocephaly, with an OFC of 37 cm (p90). All motor development milestones were delayed: he was able to sit independently at 18.5 months and to walk at 3 years and 3 months of age. He presented with language impairment. He had a severe intellectual disability. A brain MRI was performed and showed a retrocerebellar cyst. His facial dysmorphisms included brachycephaly, synophrys, epicanthus, small mouth, and pointed chin. Physical examination revealed global hypotonia, pectus excavatum, joint laxity, short fingers, and pes planovalgus. Previous metabolic and genetic investigations, including extensive metabolic screening, chromosome analysis, array CGH, Fragile X Syndrome testing (FMR1), intellectual disability panel, and trio ES, had returned normal results. GS led to the identification of a *de novo* complex rearrangement involving chromosomes 6 and 11 (Figure 4).

#### 3.5.5 Individual 5—*SPTAN1*

Individual 5 was a 23-year-old man, the third child of unaffected, consanguineous Algerian parents. The pregnancy had been uneventful. He was born at 41 WG with normal birth length (53 cm, p85), weight (3520 g, p43), and OFC (35.5 cm, p55). He had severe gastroesophageal reflux requiring Nissen fundoplication. He had limited acquisition of motor skills for his age: he walked at 18 months. He presented with delayed speech and language development followed by severe intellectual disability. Brain MRI was normal. Physical examination revealed no obvious dysmorphic features, microcephaly (−2.5 SD), slender build, high palate, hypermobile finger joints, and myopia. He experienced attention deficit hyperactivity disorder. Previous genetic investigations, consisting of array CGH and trio ES, had returned normal results. GS detected no obvious anomalies. RNA-seq evidenced a splicing event in *SPTAN1*. This RNA splicing alteration consisting of exon skipping was validated by visual inspection of the RNA-seq alignment and then of the genome sequencing alignment in IGV. This SV of ∼11 kb was associated with breakpoints at AluSx elements flanking the deleted region and was *de novo* (Figure 3). The *SPTAN1* gene is responsible for a broad spectrum of neurodevelopmental phenotypes characterized by moderate intellectual disability, with or without epilepsy and behavioral disorders (Syrbe et al., 2017). Reverse phenotyping was consistent with developmental and epileptic encephalopathy-5 (MIM 613477).

## 4 Discussion

Thirty individuals with malformation syndromes and/or severe neuro-developmental disorders and negative first-line trio ES were recruited from four centers in France. Short-read GS is becoming more affordable compared to other next-generation sequencing-based genomics technologies in diagnostics settings. In our study, the main explanation for the diagnostic yield of GS was the identification, with higher sensitivity, of genomic variations in coding and non-coding regions, such as indels (small insertion-deletions) not enriched by ES, copy-number variations (CNVs), and complex structural chromosomal rearrangements (Gilissen et al., 2014; Belkadi et al., 2015; Boycott et al., 2019; Burdick et al., 2020). Unbalanced structural variants below the detection limit of comparative chromosomal hybridization techniques are probably underdiagnosed in Mendelian disorders. GS represents a good candidate to overtake array CGH in the future, although identifying structural variants from NGS data still represents a challenge for bioinformatics (Mahmoud et al., 2019; Kobren et al., 2021). The use of GRCh38, which can be a better reference than GRCh37, can improve SV detection although it is not routine (Guo et al., 2017; Pan et al., 2019; Wagner et al., 2022). However, in our study, reanalyzing sequencing data using the GRCh38 reference genome did not lead to further diagnoses. We expect the use of the latest reference genome, obtained from the Telomere-2-Telomere consortium (Nurk et al., 2022) the optimization of bioinformatics pipelines, and the implementation of long-read sequencing technology and optical mapping approaches to improve CNV/SV detection (Chaisson et al., 2015; Chan et al., 2018; Logsdon et al., 2020). Finally, GS is far from being considered a comprehensive method to detect all types of genetic variants (mosaic variants, for example, require very deep sequencing of target regions) or to interpret the clinical implication of deep intronic variants (Boycott et al., 2019). In this respect, the integration of RNA-seq data is essential because they can identify variations in RNA abundance and sequence (i.e., gene expression outliers, allele-specific expression, splicing aberrations, and gene fusions). Thus far, several computational approaches have been developed either for transcript abundance or differential splicing (Cummings et al., 2020; Mehmood et al., 2020; Shahjaman et al., 2020). Moreover, integrating ES or GS and transcriptome analyses has shown an increased diagnostic yield of 7.5%–35% depending on the tissue analyzed and the homogeneity of the disease studied (Kremer et al., 2018, 2017; Cummings et al., 2017; Frésard et al., 2019; Gonorazky et al., 2019; Hamanaka et al., 2019; Lee et al., 2020; Murdock et al., 2020; Stenton and Prokisch, 2020; Yépez et al., 2022). In our study, RNA-seq was performed on 23 out of 30 individuals with a combined diagnostic yield of 17.4% including the identification of one structural variant not detected by GS alone, the confirmation of an intronic variant of unknown significance observed by GS, and the confirmation of two causal variants identified by GS. Of note, it was possible to confirm the pathogenic role of the intronic *TCF4* variant due to the availability of a fibroblast cell line, utilized as a second-tier approach after RNA-seq in blood. This result, together with the failure to validate the effect of the *de novo* inversion in *MEF2C* regulatory region and the deep intronic *GRIN2B* variant, emphasizes the need to perform RNA-seq in clinically accessible samples that adequately represent splicing events in relevant but non-accessible tissues (Aicher et al., 2020). Often, clinically accessible tissues deployed in these studies are blood, skin, or muscle biopsies (e.g., whole blood, Epstein-Barr virus-transformed lymphocytes, fibroblasts, and myocytes). The expression of *MEF2C* in the brain is controlled by tissue-specific regulatory elements, and perturbation of their activity cannot be modelled in peripheral tissues. To overcome these limitations, iPS-derived cell lines are sometimes used to obtain a more suitable tissue for further analysis. In most cases, RNA-seq derived from fibroblasts exhibits higher and less variable gene expression in clinically relevant genes, as Murdock et al. showed in their cohort of 115 undiagnosed patients with diverse phenotypes (Murdock et al., 2020). Furthermore, RNA-seq allowed us to exclude one candidate variant, preventing a misdiagnosis.

Finally, using DNA methylation episignatures, which are highly sensitive and specific DNA methylation biomarkers, can result in the diagnosis of rare neurodevelopmental disorders (Aref-Eshghi et al., 2020, 2019; Sadikovic et al., 2021; Levy et al., 2022), allowing VUS in genes with an established episignature to be assessed or reclassified. In our analysis, DNA methylation corroborated one patient’s molecular diagnosis of Kabuki syndrome. In another patient with a clinical diagnosis of Coffin-Siris syndrome, it found a positive episignature for a BAFopathy. However, he had negative GS and RNA-seq results, apart from a variant of unknown significance in *FGD1*, which was excluded from involvement following examination of its specific episignature. Further analyses will be required to identify the associated causal variants, including RNA sequencing using patient-derived fibroblasts and long-read sequencing or optical genome mapping. DNA methylation found also found the episignature for Wolf-Hirschhorn syndrome (WHS) in an individual with a *de novo* heterozygous *ARID5* variant. Further studies will be required to investigate the extent to which ARID5B shares differentially methylated regions with WHS. Moreover, our findings in two individuals were inconclusive. These results might be due to fewer penetrant variants, interference from a yet to be defined episignature or technical artifact.

Overall, the combined diagnostic yield of GS, RNA-seq, and DNA methylation analysis in our approach was 33.3%. We identified strong candidate variants for 13.3% additional patients that will require further functional validation. We expect the deployment of new bioinformatics pipelines for detecting SV/CNV, mobile element insertions or mitochondrial DNA genome variants (Garret et al., 2019; Niu et al., 2022) in combination with the development of new disease-associated episignatures and the advent of third generation genome sequencing or optical mapping to improve the identification of pathogenic genetic variants.

## Data Availability

The datasets presented in this study can be found in online repositories. The names of the repository/repositories and accession number(s) can be found below: ClinVar accession numbers: VCV000827810.2, SUB12094393, SUB12094652, VCV001708019.1, VCV001708028.1.

## Supplementary material

SUPPLEMENTARY FIGURE S1 Illustration of TCF4 findings in individual 30. **(A)** Ideogram showing chromosome 18 and TCF4 localization. **(B)** IGV (integrative genomics viewer) visualization of GS results showing a *de novo* heterozygous deep intronic variant in *TCF4*. **(C)** Sashimi plot visualization showing the inclusion of a cryptic exon (chr18:g.52926121-52926348), predicted to result in a frameshift variant p.Ala357Glyfs*7. **(D)** IGV visualization of RNA-seq data in individual 30 and an unaffected control showing the monoallelic expression of an informative single nucleotide polymorphism located in the 3′ end of TCF4.

SUPPLEMENTARY FIGURE S2 EpiSign (DNA methylation) MVP scores from this cohort. A multi-class supervised classification system capable of discerning between multiple episignatures by generating a probability score (MVP) for each episignature. A positive score is typically greater than 0.5, and three patients produced an MVP of 1.0, indicating a methylation profile match for BAFopathy (red), Kabuki syndrome (green), and Wolf-Hirschhorn syndrome (purple). Two cases (dark grey) were inconclusive for Rubinstein-Taybi syndrome and Velocardiofacial syndrome as MVP elevation was insufficient. All remaining cases (light grey) were negative for all 59 episignatures analyzed.

SUPPLEMENTARY FIGURE S3 DNA methylation analysis of BAF complex gene promoters. DNA methylation analysis of promoter regions in individual 11 for **(A,B)** ARID1A **(D,D)** ARID1B **(E–G)** SMARCA2 **(H,I)** SMARCA4 **(J)** SMARCB1. All of them were within normal range.

SUPPLEMENTARY TABLE S1 Phenotype and genotype of all 30 individuals in the OMIXCARE cohort. F, female; M, male; ID, intellectual disability; N, no; Y, yes; NA, not available; /, absent; m, months; y, years; W, weight; H, height; HC, head circumference; EEG, electroencephalogram. Dark green signifies the identified genes, light green is for the candidate genes, and orange is for the rejected genes.
