# Supplementary figures and images for: OMIXCARE: OMICS technologies solved about 33% of the patients with heterogeneous rare neuro-developmental disorders and negative exome sequencing results and identified 13% additional candidate variants
Source: Front Cell Dev Biol. 2022 Oct 28;10:1021785. doi: 10.3389/fcell.2022.1021785 (PMC9650323; doi:10.3389/fcell.2022.1021785)

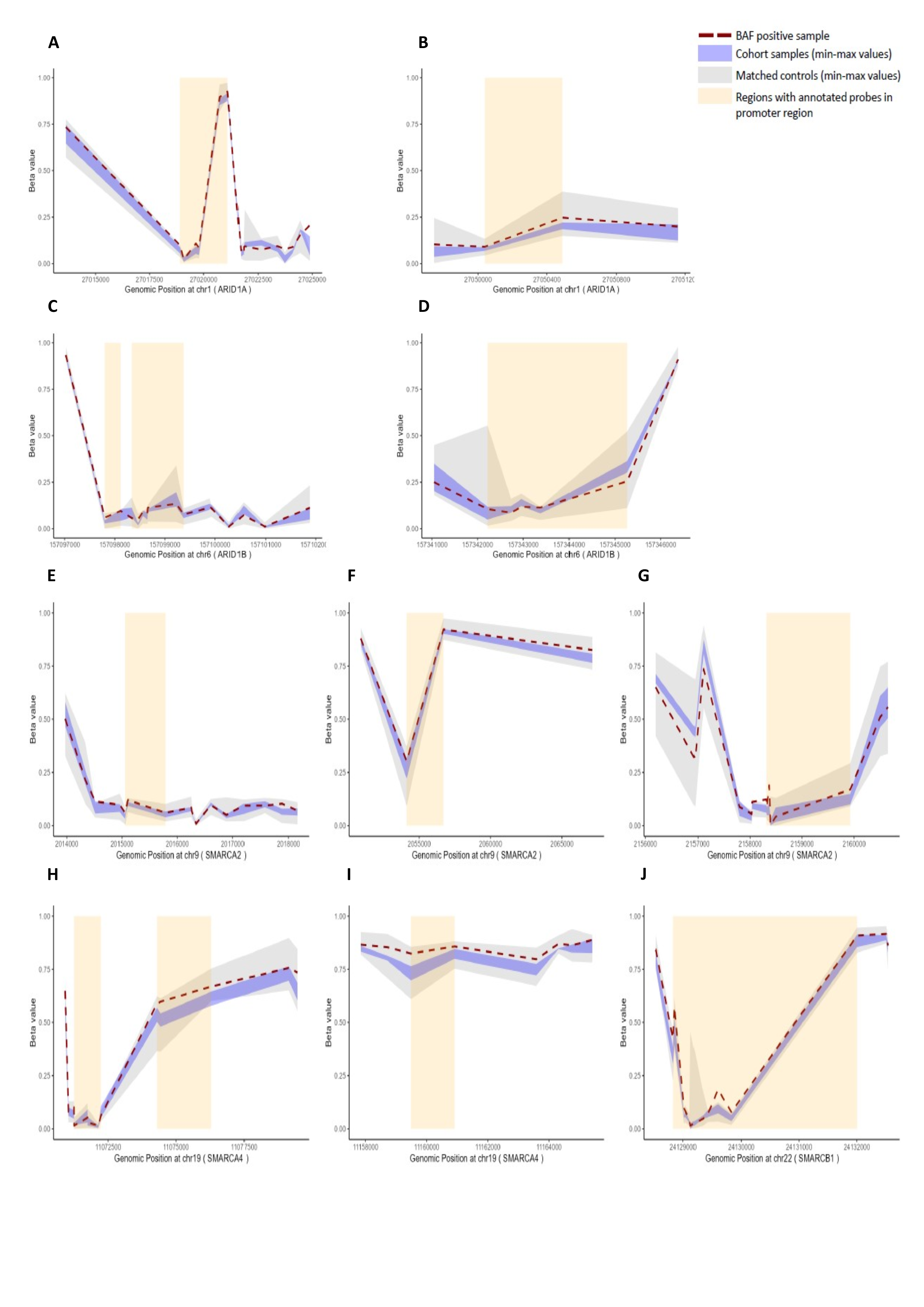

Supplement: Supplementary file 1 [file Image3.TIFF]

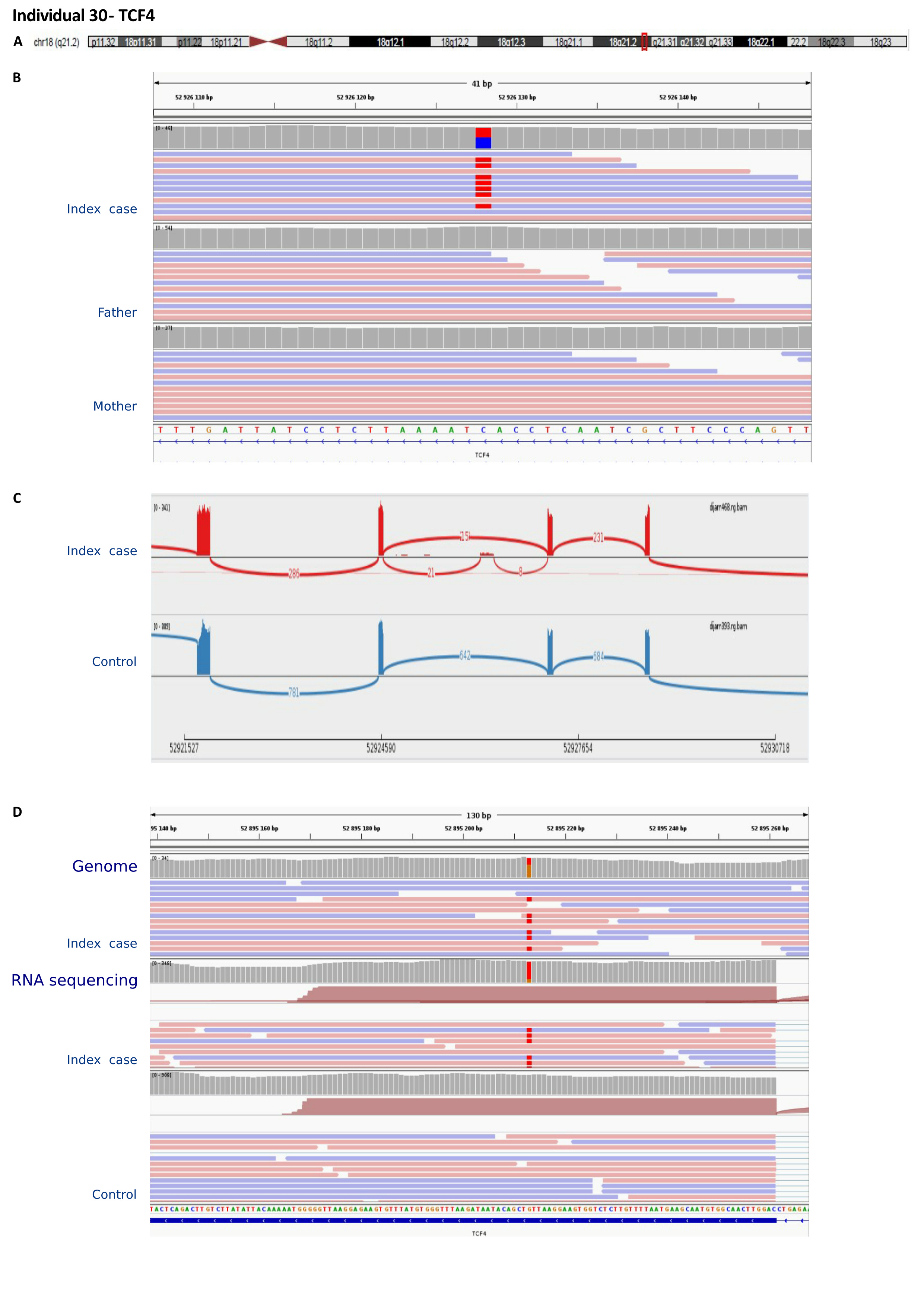

Supplement: Supplementary file 2 [file Image1.TIF]

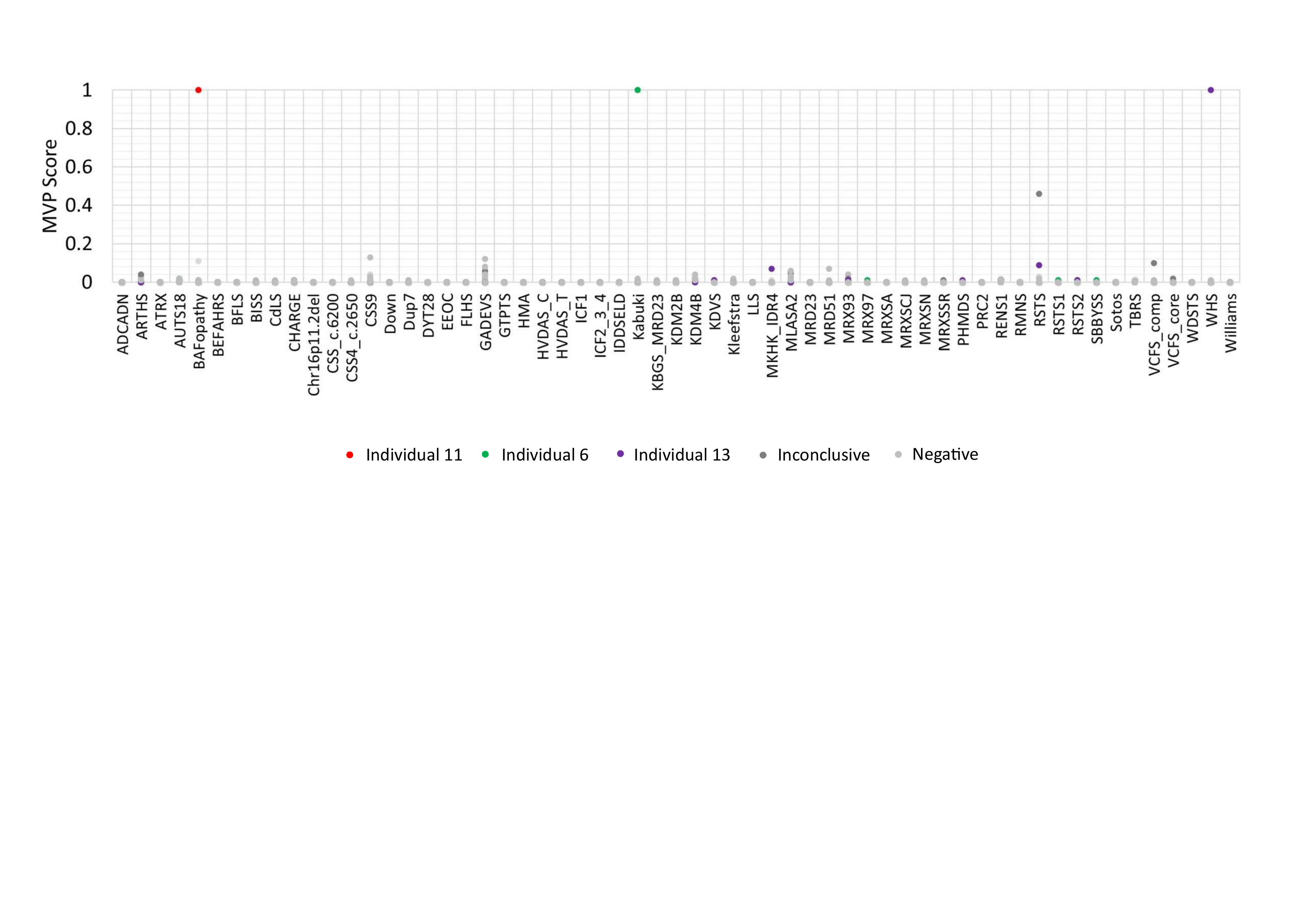

Supplement: Supplementary file 4 [file Image2.TIFF]
