# Supplementary material for: OMIXCARE: OMICS technologies solved about 33% of the patients with heterogeneous rare neuro-developmental disorders and negative exome sequencing results and identified 13% additional candidate variants
Source: Front Cell Dev Biol. 2022 Oct 28;10:1021785. doi: 10.3389/fcell.2022.1021785 (PMC9650323; doi:10.3389/fcell.2022.1021785)
Supplement: Supplementary file 5 [file DataSheet1.docx]

**Supplementary Clinical information**

**Positive results**

***Individual 6 # KMT2D***

Individual 6 is a 5-year-old girl, the second child of unaffected, non-consanguineous French parents. The pregnancy had been uncomplicated with normal screening ultrasounds. She was born at 39 weeks of gestation (WG) with normal birth length (48 cm, p27), weight (3180 g, p48), and OFC (33.5 cm, p32). A cleft palate and a congenital bilateral hip dislocation were noted at birth. During the neonatal period, she underwent palate surgery. She demonstrated poor feeding. At the echocardiography, a ventricular septal defect and an abnormal mitral valve morphology were noted. All milestones of motor development were delayed: she was able to sit independently at 13 months and to walk at 2 years and 6 months of age. She had delayed speech and language development. She has facial dysmorphisms including upslanted palpebral fissures, blue sclerae, and gingival overgrowth. Examination of the extremities showed persistence of prominent fingertip pads and pes talus. Array CGH and trio ES were performed with the hypothesis of Kabuki syndrome, but results were normal. GS led to the identification of a *de* *novo* nonsense variant p.(Gln3964*) in *KMT2D,* classified as ACMG class 5. The *KMT2D* gene is involved in Kabuki syndrome.

***Individual 7 # FOXG1***

Individual 7 is an 8-year-old girl, the only child of unaffected, non-consanguineous French parents. The pregnancy has been marked by gestational diabetes. She was born at 41 WG+1 by cesarean section because of altered fetal heart rate with intrauterine growth retardation; birth length was 46.5 cm (p1), weight 2985 g (p11) and OFC 33.5 cm (p19). She had neonatal hypotonia with limb hypertonia. She demonstrated poor feeding. All milestones of motor development were severely delayed: she was able to sit independently at 2 years, but is still unstable. She cannot walk at 8 years of age. She has language impairment. At 2 years of age she started to present with complex febrile seizures and tonic seizures. She has acquired microcephaly (<-4 SD). A brain MRI showed hyperintensity of the caudate nucleus on T2-weighted images and a parahippocampal gyrus of small volume. Physical examination revealed no obvious dysmorphic features, except strabismus. She has experienced sleep disturbance, and has shown motor stereotypies and paroxysmal bursts of laughter. Previous genetic investigations, consisting of array CGH, screening for Rett syndrome (*MECP2*) and Angelman syndrome (methylation of the *UBE3A*), intellectual disability and autism spectrum panel, and trio ES, produced normal results. GS led to the identification of a *de* *novo* frameshift variant p.(Gln86fs) in *FOXG1,* classified as ACMG class 5. The *FOXG1* gene is involved in the congenital variant of Rett syndrome, which is a severe neurodevelopmental disorder with earlier onset features than those of classic Rett syndrome.

***Individual 8 # PURA***

Individual 8 is a 7-year-old girl, the second child of unaffected, non-consanguineous French parents. On the maternal side, she has two uncles, with one cousin who has an intellectual disability due to an *OPHN1* alteration. The mother of individual 3 has been tested and she is not a career of this alteration. The pregnancy was uncomplicated with normal screening ultrasounds. She was born at 42 WG and was eutrophic at birth. She presented with neonatal hypotonia, poor eye contact and poor feeding. All milestones of motor development were delayed: she was able to sit independently at 23 months, but she cannot walk independently at 7 years of age. She has language impairment. A brain MRI at the age of 18 months was normal. Physical examination revealed no obvious dysmorphic features, except lumbar scoliosis. She presents with motor stereotypies. Ophthalmologic examination showed abnormal visual electrophysiology. Previous metabolic and genetic investigations, consisting of analysis of neurotransmitter levels in cerebrospinal fluid, folate level, urine mucopolysaccharides, urine oligosaccharides, array CGH, and trio ES, produced normal results. GS led to the identification of a *de* *novo* frameshift variant p.(Gly34fs) in *PURA*, classified as ACMG class 5. The *PURA* gene is involved in PURA syndrome, which is characterized by a moderate to severe degree of learning disability and developmental delay, seizures, feeding difficulties, respiratory insufficiency, abnormal vision, and hypersomnolence.

***Individual 9 # CYFIP2***

Individual 9 is a 14-year-old girl, the only child of unaffected, non-consanguineous parents of French/North African descent. The pregnancy was uncomplicated. She was born at 37 WG with normal birth length (46.5 cm, p31), weight (2800g, p50), and OFC (34.5 cm, p81). Axial hypotonia was noted at 4 months of age. She demonstrated poor feeding. All milestones of motor development were delayed: she was able to sit independently at 9 months and to walk at 3 years of age. She has delayed speech and language development and she still uses both verbal and nonverbal communication. A brain MRI was performed twice and was normal. She has facial dysmorphisms including hypertelorism, epicanthus, long eyelashes, bulbous nose, wide mouth, gingival overgrowth, and retrognathia. Physical examination revealed poor overall growth (length -2 SD, weight -2 SD), pes planus, joint hyperlaxity, and lumbar hyperlordosis. She is described as a shy girl. She displays auto- and hetero-aggressive behavior. She has experienced sleep disturbance from the age of 10. Previous genetic investigations, consisting of chromosome analysis, array CGH and trio ES, produced normal results. GS led to the identification of a *de* *novo* missense variant p.(Asp699Gly) in *CYFIP2,* classified as ACMG class 5. The *CYFIP2* gene is involved in developmental and epileptic encephalopathy-65 (DEE65).

***Individual 12 # TMEM147***

Individual 12 is a 16-year-old girl, the first child of unaffected, non-consanguineous French parents. The pregnancy was marked by placental abruption and preterm labor. She was born at 40 WG by emergency cesarean section due to fetal distress and meconium in the fluid. She had eutrophic birth length (52 cm, p90), weight (3020g, p20), and OFC (33 cm, p13). The neonatal period was marked by breathing problems, poor feeding and bilateral ovarian hernias. All milestones of motor development were delayed: she was able to sit independently at 10.5 months and to walk at 21 months of age. She has language impairment. A brain MRI at the age of two showed ventricular enlargement. A second brain MRI at the age of 11 was normal. She has facial dysmorphisms including posterior plagiocephaly, prominent forehead, hypertelorism, strabismus, smooth and long philtrum, and wide mouth. Physical examination revealed abnormal hair pigmentation and texture, clinodactyly of the 5th finger, pes planus, and scoliosis. Neurological examination showed generalized hypotonia, lower limb spasticity, toe walking, and oral-motor apraxia. She has attention deficit hyperactivity disorder, stereotypy and displays abnormally aggressive, impulsive or violent behavior. She has experienced sleep disturbance. Previous metabolic and genetic investigations, consisting of serum amino acids, urine organic acids, serum lactate, plasma and urine creatine and guanidinoacetate, very long chain fatty acids, phytanic acid, urine mucopolysaccharides and oligosaccharides, transferrin isoform analysis for congenital disorders of glycosylation, chromosome analysis, array CGH, Fragile X Syndrome testing (FMR1), RASopathies panel, screening for Smith-Magenis syndrome (*RAI1*), and trio ES, all produced normal results. GS led to the identification of two compound heterozygous variants, a paternally inherited frameshift variant p.Lys34fs, and a maternally inherited nonsense variant p.(Tyr162*) in *TMEM147,* both classified as ACMG class 3.

**Candidate gene**

***Individual 10 # POLA1***

Individual 10 is a 35-year-old male, the second child of unaffected, non-consanguineous French parents. The perinatal period was marked by hypoxia because of umbilical cord coiling. He presented mild global developmental delay: he was able to walk at 20 months of age. He has apraxia. He began presenting with seizures from the age of four. He has facial dysmorphisms including dolichocephaly with microcephaly, low anterior hairline, long eyelashes, synophrys, thin eyebrows, epicanthus, and large ears. Physical examination revealed tall stature, lower limbs of unequal length, quadriceps atrophy, genu valgum, brachydactyly type E, prominent fingertip pads, pectus excavatum, and thoracic kyphosis. He experienced precocious puberty, spinal cord compression due to an arteriovenous malformation, recurrent dental infections, trigeminal neuralgia, and osteoporotic vertebral compression. A brain MRI at 31 years of age showed vascular loops of the left internal carotid and the left vertebral artery resulting in impingement of the left trigeminal nerve. A diagnosis of Kabuki syndrome was evoked in infancy. Previous genetic investigations, consisting of chromosome analysis, array CGH and trio ES, produced normal results. GS led to the identification of an inherited missense variant p.(Lys1432Ile) in *POLA1,* classified as ACMG class 3.

***Individual 11 # BAFopathy***

Individual 11 is a 15-year-old boy, the third child of unaffected, non-consanguineous French parents. The pregnancy was uncomplicated. He was born at 39 WG +3 with normal birth length (49 cm, p31) and weight (3240g, p40). During the neonatal period, he had recurrent chronic otitis media and respiratory infections. All milestones of motor development were delayed: he was able to sit independently at 16.5 months and to walk at 3 years and 6 months of age. He has language impairment. A brain MRI showed an arachnoid cyst and partial posterior agenesis of the corpus callosum. He has sensorineural hearing loss, hypermetropia, and photophobia. He has facial dysmorphisms including brachycephaly, frizzy hair, low hairline posteriorly rotated ears, and sparse eyebrows. Physical examination showed bilateral hypoplasia of the 3rd phalanges of the 5th fingers, dorsal hypertrichosis, and decreased muscle strength in the lower limbs. He has feeding difficulties. He displays bruxism, stereotypy, and a short attention span. Previous genetic investigations, consisting of array CGH and trio ES, produced normal results. GS first led to the identification of a maternally inherited missense variant p.(Arg449Cys) in *FGD1,* classified as ACMG class 3. The *FGD1* gene is involved in an X-linked intellectual disability syndrome. A segregation study highlighted the presence of the same variant in the maternal first cousin of the propositus, who presents slight difficulties in language acquisition associated with a relatively small stature, microcephaly (-2 SD), and some dysmorphic features. Episignature analysis refuted this hypothesis and showed individual 11 to have the BAF signature. Reanalysis of GS and RNA-seq did not find evidence of SNP or SV in the BAFopathy genes. A new blood sample for optical mapping and fibroblast cell culture for RNA-seq should improve the identification of a pathogenic variant.

***Individual 13 # ARID5B***

Individual 13 is a 7-year-old girl, the second child of unaffected, non-consanguineous French parents. The pregnancy was marked by oligoamnios. She was born at 38 WG with intrauterine growth retardation, birth length 47 cm, (p24), weight 2380g (p4), and OFC 36cm (p95). The neonatal period was marked by a congenital hip dislocation, pes valgus, and broad neck. All milestones of motor development were delayed: she was able to sit independently at 11 months and to walk at 30 months of age. She has delayed speech and language development. She has facial dysmorphisms including prominent forehead, wide mouth, retrognathia, oligodontia, strabismus, and blue sclerae. She has severe intellectual disability. Previous genetic investigations, consisting of array CGH and trio ES, produced normal results. GS led to the identification of a *de* *novo* frameshift variant p.(Asn434fs) in *ARID5,* classified as ACMG class 3. Interestingly, DNA methylation found an episignature suggestive of Wolf-Hirschhorn Syndrome in this individual.

***Individual 14 # GRIN2B***

Individual 14 is a boy aged 4 years and 6 months, the third child of unaffected, non-consanguineous French parents. The pregnancy was uncomplicated. He was born at 41 WG with eutrophic birth length (50 cm, p28) and weight (3500g, p41). He had hypotonia and delayed motor development: he was able to sit independently at 12 months and to walk with an ataxic gait at 22 months of age. He started to present with myoclonic epilepsy at age 9 months, controlled by sodium valproate. His brain MRI is normal. He has an intellectual disability. He has language impairment and uses nonverbal communication. Physical examination revealed no obvious dysmorphic features. He presents motor stereotypies. Previous metabolic and genetic investigations, consisting of array CGH, screening for Angelman syndrome (methylation of the UBE3A), intellectual disability panel, and trio ES, produced normal results. GS led to the identification of a *de* *novo* deep intronic variant NM_000834.3:c.1010+13168T>C in *GRIN2B*, classified as ACMG class 3. The *GRIN2B* gene is involved in intellectual developmental disorder with or without seizures. RNA-seq on blood samples was inconclusive because of neuronal tissue-specific expression. RNA-seq will be performed on induced neural stem cells.

***Individual 15 # SENP6***

Individual 15 is a 17-year-old girl, the first child of four with an affected brother. Her parents are consanguineous. The pregnancy was uncomplicated. She was born at 41 WG with normal birth weight (3200g, p27) and OFC (35cm, p61). She has an intellectual disability with delayed fine motor development and expressive language. She has demonstrated behavioral abnormality with psychotic mentation. She has facial dysmorphisms including small forehead, prominent metopic ridge, synophrys, epicanthus, and anteverted nares. Physical examination revealed tall stature, high palate, and strabismus. Her brain MRI and EEG were normal. Previous metabolic and genetic investigations, consisting of serum amino acids, urine organic acids, serum lactate, plasma and urine creatine and guanidinoacetate, creatine kinase, chromosome analysis, array CGH, Fragile X Syndrome testing (*FMR1*), and trio ES, produced normal results. GS led to the identification of a homozygous nonsense variant p.(Arg157*) in *SENP6,* classified as ACMG class 3. The same genotype was found in her brother who also has psychomotor retardation. However, RNA-seq did not confirm a transcriptome outlier detection of *SENP6*.

**Legends to Table S1: Phenotype and genotype of all 30 individuals in the OMIXCARE cohort**

F: female, M: male, ID: intellectual disability, N: no, Y: yes, NA: not available, /: absent, m: months, y: years, W: weight, H: height, HC: head circumference, EEG: electroencephalogram. Dark green signifies the identified genes, light green is for the candidate genes, and orange is for the rejected genes.,
